# Development of the E-Nabız Usage Behavior Scale: Validity and Reliability Study

**DOI:** 10.3390/healthcare14172757

**Published:** 2026-08-31

**Authors:** Beyza Ünlü Büyükalim, Selma Pekgör, Fatih Yılmaz

**Affiliations:** 1 Department of Family Medicine, Konya City Hospital, 42020 Konya, Türkiye; 2 Ministry of National Education, 42020 Konya, Türkiye

**Keywords:** digital health, e-Nabız, reliability, scale development, validity

## Abstract

**Highlights:**

**What are the main findings?**

- The 6-item e-Nabız Usage Behavior Scale (eNUBS) was developed as a unidimensional, structurally valid, and internally consistent instrument to measure real-world personal health record utilization.
- Psychometric analyses demonstrated acceptable construct reliability (*α* = 0.72), stable factor loadings (0.59 to 0.76), and adequate model fit indices via confirmatory factor analysis.

**What are the implications of the main findings?**

- The eNUBS provides an ultra-brief, practical screening tool for clinicians and researchers to objectively assess and monitor patient engagement with national digital health portals.
- The scale serves as an adaptable global prototype that can be cross-culturally modified to evaluate user behaviors toward emerging digital healthcare infrastructures worldwide.

**Abstract:**

**Objective**: This study aims to evaluate the validity and reliability of the “e-Nabız Usage Behavior Scale” (eNUBS), developed to measure user behaviors toward Türkiye’s national personal health record system. **Methods**: This methodological study included 304 voluntary adult, non-healthcare participants at a hospital. Data were collected via an 8-item sociodemographic form and a 6-item draft scale. Construct validity was evaluated using Exploratory Factor Analysis (EFA) and Confirmatory Factor Analysis (CFA); reliability was assessed using Cronbach’s alpha and item-total correlations. **Results**: Participants were 61.8% male, with a mean age of 37.79 ± 9.40. EFA confirmed a unidimensional structure, explaining 46.21% of the total variance. The Cronbach’s alpha was 0.72, and corrected item-total correlations ranged from 0.43 to 0.59. CFA demonstrated adequate model fit indices. **Conclusions**: The eNUBS is a brief, valid, and reliable tool for assessing real-world digital health portal usage, offering valuable guidance for digital health policy and for monitoring patient engagement.

## 1. Introduction

The integration of digital tools has evolved from a supplemental feature into a transformative force, reshaping the fundamental mechanics of both clinical delivery and longitudinal patient engagement. These systems enable individuals to securely access their health data via the internet or mobile applications, thereby supporting more active participation in health processes. Patient portals, in particular those that provide access to personal health records, have the potential to increase patient participation in health management through functions such as viewing laboratory results, tracking prescriptions, and making appointments [1].

The literature indicates that patient portals can strengthen doctor–patient communication, increase patient satisfaction, and improve health outcomes [1]. However, the key factor determining the effectiveness of these systems is not just technical access but how frequently and for what purposes users use the portals in real-world settings. Therefore, the success of digital health systems depends directly on users’ behavioral adoption of these tools.

Knowledge level is a key determinant of the use of digital health systems. For example, portal usage rates have been reported to be significantly lower among individuals with low digital health literacy [2]. In contrast, even with a high level of knowledge, factors such as security concerns and lack of motivation can limit active use [3,4,5].

The e-Nabız system, developed in Türkiye, is a comprehensive national digital health portal that provides individuals with access to their health data and a wide range of integrated health services [6]. Studies conducted in Türkiye have provided significant findings on the usability of e-Nabız and user perceptions; the system’s technical features and user experience have been evaluated using qualitative and quantitative methods [7,8]. However, the vast majority of existing studies examine users’ perceptions or satisfaction levels; studies focused on developing a valid and reliable behavior-based measurement tool to directly measure e-Nabız usage remain limited.

The e-Nabız system, developed in Türkiye, is a comprehensive national digital health portal that provides individuals with access to their health data and a wide range of integrated health services. Studies conducted in Türkiye have provided significant findings on the usability of e-Nabız and user perceptions; the system’s technical features and user experience have been evaluated using qualitative and quantitative methods. However, the vast majority of existing studies examine users’ perceptions or satisfaction levels; studies focused on developing a valid and reliable behavior-based measurement tool to directly measure e-Nabız usage remain limited. Although existing research has widely evaluated user satisfaction and system usability, tools designed to directly quantify real-world behavioral frequency and adoption patterns are scarce. Therefore, developing a short, practical, and psychometrically valid scale to objectively assess the real-world use of digital health systems addresses this clear methodological gap for both the scientific literature and clinical practice.

## 2. Material & Method

### 2.1. Research Design

This methodological study was designed to develop and psychometrically evaluate a brief, practical, and objective instrument to measure the real-world usage behaviors of individuals interacting with digital health portals. Using a cross-sectional survey design, the research systematically assessed the structural validity, internal consistency, and construct reliability of the newly developed scale in a sample of active healthcare service consumers.

### 2.2. Population & Sample

The study population comprised 304 individuals who participated voluntarily and visited the Konya City Hospital Department of Family Medicine. A total of 309 patients visiting the family medicine outpatient clinic were informed about the purpose of the study. Of these, 304 patients agreed to participate in the study. Five patients refused to participate due to limited time or unwillingness to provide information. Accordingly, the participation rate is around 98%. Based on the criterion of “a sample size at least 5–10 times the number of items” recommended in scale development studies, the sample size for the 6-item scale was considered sufficient [9].

The study’s inclusion criteria are as follows:

1. Having applied to the hospital for an examination;
2. Agreeing to participate in the study;
3. Being at least 18 years old.

On the other side, the study’s exclusion criteria are as follows:

1. Being a healthcare worker;
2. Not understanding Turkish or being unable to communicate;
3. Lacking the cognitive ability to answer questions;
4. Having any physical (hearing or speech impairment), neurological, or mental disorders (intellectual disability or psychotic disorder) that would affect completion of the forms.

### 2.3. Data Collection Tools

Data were collected using the “Sociodemographic Data Form,” consisting of 8 researcher-developed questions, and the draft “E-Nabız Usage Behavior Scale,” also developed by the researchers, to measure e-Nabız use-related behavior.

The sociodemographic data form includes questions about individuals’ age, gender, educational background, marital status, perceived income level, employment status, the institution where they most frequently receive healthcare services, and the presence of chronic diseases.

The scale items were developed based on a literature review of the e-Nabız system, digital health literacy, patient portals, and personal health record systems. A preliminary study was conducted with 25 individuals who shared characteristics with the sample group to assess the comprehensibility of the draft scale items prior to data collection. The pilot study group was not included in the sample group. After the pilot study, no unclear expressions were observed.

### 2.4. Content Validity

To establish content validity, the draft item pool was evaluated by an expert panel. Initially, an item pool of 6 items was generated based on a literature review regarding digital health portals and user behaviors. This pool was reviewed by an expert panel consisting of 4 specialists (3 in Family Medicine and 1 in Public Health) to assess clarity, relevance, and representativeness. Based on content relevance, clarity and comprehensibility, the items which were deemed appropriate and relevant to the intended construct were retained. Since the abovementioned experts agreed on the six proposed items, no items were excluded in this stage.

### 2.5. Scale Structure

The e-Nabız Behavior Scale is a 5-point Likert-type scale with response options ranging from “Never” to “Always”.

### 2.6. Ethical Approval

Ethical approval for the study was obtained from the KTO Faculty of Medicine Ethics Committee on 25 September 2025 (decision number 2025/060). Prior to data collection, written informed consent was obtained from each participant, and all research protocols strictly adhered to the ethical principles outlined in the Declaration of Helsinki.

### 2.7. Statistical Analysis

Data analysis was conducted using IBM SPSS version 23. To evaluate the construct validity of the scale, exploratory factor analysis (EFA) was conducted, and the suitability for factor analysis was assessed using the Kaiser–Meyer–Olkin (KMO) measure of sampling adequacy and Bartlett’s test of sphericity. Scale reliability was assessed using Cronbach’s alpha and item-total correlations. Given the brief, highly compact structure of the 6-item e-Nabız Usage Behavior Scale, both EFA and CFA were conducted on the same sample (*N* = 304) to substantiate the overall model fit and construct validity.

## 3. Results

The initial findings on participants’ demographic characteristics indicate that 304 individuals completed the data collection tool. A total of 188 participants are male (61.8%), and 116 are female (38.2%). The average age is 37.79 (±9.40), with an age range of 18–80.

The majority of participants hold a bachelor’s degree (*n* = 152; 50%), followed by postgraduate degrees (*n* = 110; 36.2%), primary and secondary school graduates (*n* = 22; 7.2%), and high school graduates (*n* = 20; 6.6%). Most participants are married (*n* = 239; 78.6%), actively employed (*n* = 250; 82.2%), and do not have any chronic illnesses (*n* = 254; 83.6%).

The institutions where participants most frequently received healthcare services were state hospitals (*n* = 169; 55.6%), family health centers (*n* = 97; 31.9%), university hospitals (*n* = 21; 6.9%), and private hospitals (*n* = 17; 5.6%), respectively.

Regarding income, the majority of participants stated that their income was equal to their expenses (*n* = 179; 58.9%), while some stated that their income was less than their expenses (*n* = 67; 22%), and others indicated that it was more (*n* = 58; 19.1%).

### 3.1. Validity & Reliability

The construct validity of the “e-Nabız Usage Behavior Scale” (eNUBS) was assessed using EFA. Before extraction, the data’s suitability for analysis was evaluated using the KMO sample adequacy and Bartlett’s test of sphericity. The analysis yielded a KMO value of 0.77, indicating that the data are well suited for factor extraction (KMO = 0.77; *χ*^2^ = 413.814; *df* = 15; *p* < 0.001) [10].

### 3.2. Structural Validity

EFA, which identifies latent structures from observed variables, was used to extract factors [11]. In this context, principal component analysis with varimax rotation was applied. To determine the number of factors, eigenvalues (*λ*) were considered, and factors with eigenvalues greater than 1 were deemed significant [12,13]. In EFA, factor loadings above 0.50 were considered significant [14]. To address potential issues with items loading highly on multiple factors, cross-loadings were examined. Communalities, which indicate the variance explained by the factors, were also examined, and values between 0.40 and 0.70 were considered moderate [15]. Factor analysis confirmed that the scale has a unidimensional structure, accounting for 46.21% of the total variance. This ratio is considered acceptable according to the literature [16].

To further validate the scale’s factor structure, using the existing dataset, principal axis factoring (PAF) and parallel analysis (PA) (based on common factors [17]) were conducted in jamovi [18]. Before analysis, data suitability was confirmed by Bartlett’s test of sphericity and KMO (*χ*^2^ = 414, *df* = 15, *p* < 0.001, KMO = 0.777).

The first factor’s eigenvalue (2.15) was substantially higher than the second (0.23), and the scree plot showed that the real-data eigenvalue curve diverged clearly from the simulated-data curve only at the first factor, with the two curves nearly overlapping from the second factor onward. Although PA suggested up to three factors, the second and third factors showed weak, inconsistent loadings on only one or two items and did not indicate a simple structure. Therefore, they were not considered meaningful in terms of content. This decision was also supported by the marked decrease in eigenvalues (from 2.15 to 0.23) and by PAF analysis conducted independently in SPSS 23 [19], which also supported a single-factor structure. Accordingly, the number of factors was fixed at 1. In the one-factor solution, all 6 items showed consistent, moderate-to-strong loadings (*λ* = 0.49–0.73), explaining 35.8% of the total variance, thereby supporting the scale’s hypothesized one-factor structure.

### 3.3. Reliability Analysis

Cronbach’s alpha (*α*) was used to assess the scale’s internal consistency [20]. In the literature, an alpha of 0.70 or higher is considered acceptable [21]. Furthermore, the corrected item-total correlation (CITC) indicates the reliability of the construct, and values above 0.40 are acceptable [22]. The instrument properties are presented in Table 1.

### 3.4. Factor Validation

The EFA results indicated a single-factor structure for the scale. To substantiate the proposed model, the researchers conducted a confirmatory factor analysis (CFA) using the maximum likelihood estimation (MLE) method in IBM SPSS AMOS v23 [19,23]. Before conducting CFA, the assumptions of MLE were tested. Skewness values ranged from −0.51 to 0.50, and Kurtosis values ranged from −1.03 to −0.18, indicating acceptable normality [24]. Moreover, multivariate normality was checked employing Mardia’s multivariate kurtosis in AMOS. The outputs revealed that the multivariate kurtosis value was 4.69 and the corresponding critical ratio (c.r.) was 4.18, suggesting that the data has multivariate normality [25,26,27]. Multivariate outliers were checked employing Mahalanobis distance (*D*^2^) and the results revealed that none of the observations met the criterion of *p* < 0.001, suggesting no severe multivariate outliers [11].

To optimize the model fit indices during the CFA, error covariances were specified between items representing closely aligned behavioral habits and clinical workflows. Specifically, error terms were correlated between mobile application usage and accessing lab reports (e5–e6), as well as between general portal logins and appointment scheduling (e1–e6). Since these items reflect highly interrelated digital healthcare practices, establishing these covariances was theoretically justified and successfully improved the overall model fit.

**Figure 1:**
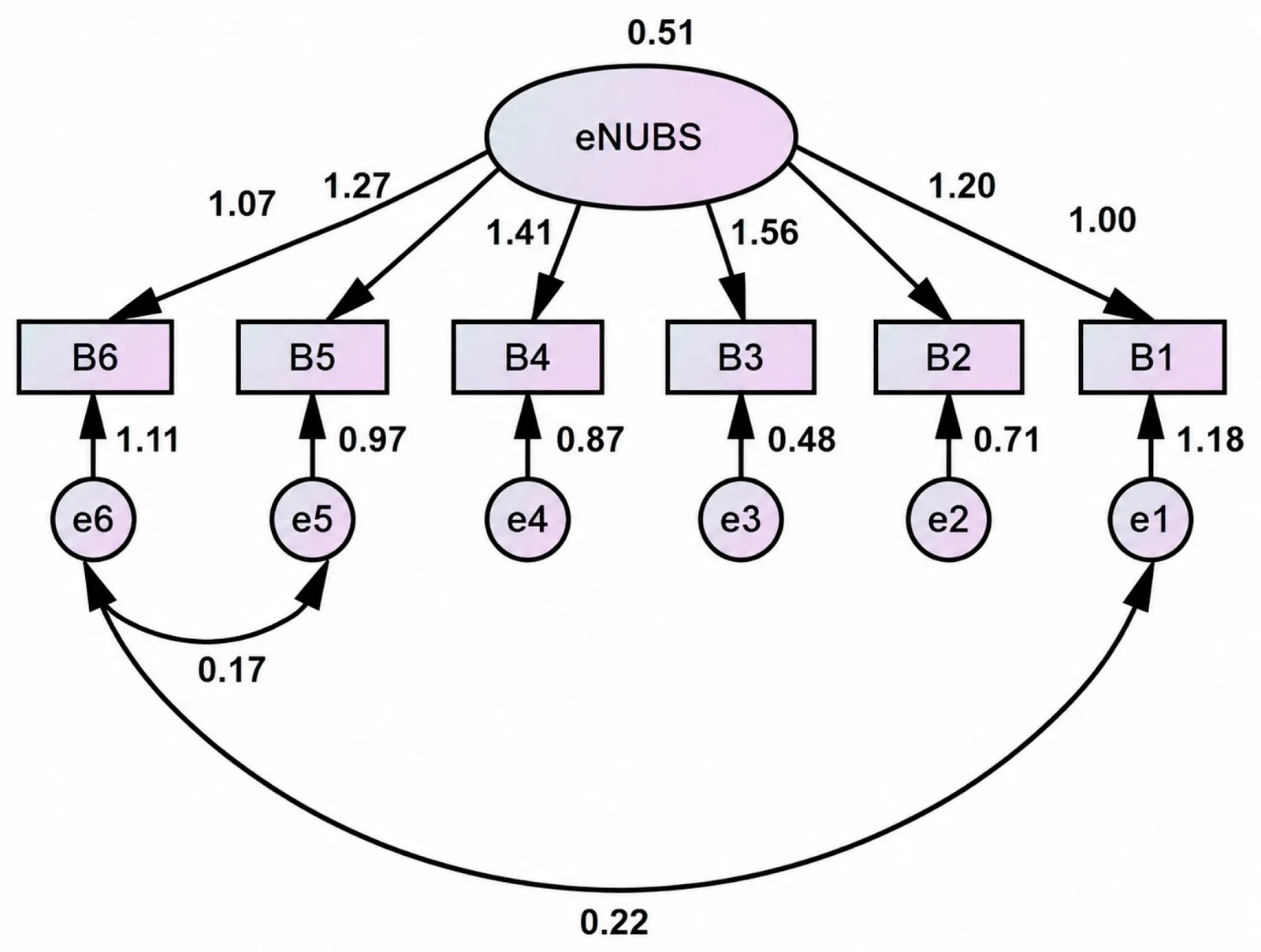
The Path Diagram.

Figure 1 presents the path diagram, and the final scale is provided in Table 2.

The CFA results showed a good fit with the data [28]. [*χ*^2^ (7, *N* = 304) = 19.691, *p* < 0.001; *χ*^2^/*df* = 2.81; RMSEA = 0.07 GFI = 0.98; CFI = 0.98; TLI = 0.96; AGFI = 0.93; NFI = 0.97; IFI = 0.98]. Moreover, standardized factor loadings ranged from 0.55 to 0.84, and all of them were statistically significant (*p* < 0.001).

## 4. Discussion

This study examined the psychometric properties of the scale developed to assess e-Nabız usage behaviors; the results indicated that the instrument is valid and reliable. In today’s world, as the integration of digital health technologies into healthcare services gains momentum, evaluating not only the presence of patient portals but also their effective use by individuals is increasingly important.

Literature reviews have shown that electronic patient portals positively affect health outcomes, patient satisfaction, and healthcare service efficiency [29,30]. However, for these benefits to materialize, users must actively use these systems. Therefore, the need for tools that measure not only user perceptions but also direct usage behavior is growing. Accordingly, the scale developed in this study makes a significant contribution to the literature by addressing this need.

In this paper, a significant portion of the participants hold undergraduate and graduate degrees. Education level is considered an important determinant of the use of digital health systems. Previous research has shown that individuals with high digital health literacy use patient portals more effectively [31]. However, there may not always be a direct correlation between knowledge level and actual usage behavior.

Research has shown that factors related to patient portal use include age, socioeconomic status, digital literacy, access to technology, and privacy concerns [32,33]. Among older age groups, limited technology use can negatively affect portal use, leading to lower engagement with digital health resources and potentially affecting health outcomes [34]. These findings indicate that in the dissemination of digital health systems, both the technical infrastructure and user behaviors should be taken into account.

The e-Nabız system, developed in Türkiye, is a comprehensive digital health platform that provides individuals with access to their health data. However, most existing studies focus on user satisfaction, perceptions, and usability; tools that measure actual usage behavior remain limited, hindering a comprehensive understanding of how users interact with digital health systems. In this context, the developed scale fills an important gap in the literature by measuring e-Nabız usage at the behavioral level. By shifting the focus from passive perceptions to active behavioral frequency, this instrument provides a distinct methodological advantage for digital health evaluation. While e-Nabız represents Türkiye’s national digital health infrastructure, the scale developed in this study serves as a global prototype for evaluating user behaviors toward Personal Health Record (PHR) systems that are expanding worldwide, such as the NHS App in the UK or My Health Record in Australia. In this regard, the core framework of the scale can be readily adapted to assess usage patterns of diverse national health portals across different countries, thereby facilitating cross-cultural comparative research.

The factorial structure derived from the validity analyses falls within acceptable limits, and the confirmatory factor analysis results indicate good fit indices, reinforcing the scale’s structural validity. Furthermore, the specification of error covariances during the CFA process was structurally and behaviorally justified. The correlation between the error terms for mobile device application usage (e5) and for accessing laboratory/test reports (e6) reflects the clinical reality that contemporary users predominantly use mobile health applications to quickly check diagnostic results on the go. Similarly, the covariance between general platform login (e1) and appointment scheduling (e3) underscores that scheduling medical visits remains one of the primary behavioral drivers prompting individuals to access national digital health portals. Addressing these shared variances significantly optimized the model’s structural configuration without compromising its theoretical foundation. Specifically, the total explained variance (46.21%) and the internal consistency coefficient (*α* = 0.72) of the scale fall well within acceptable scientific thresholds. In the psychometric literature, brief screening instruments with a limited number of items (such as this 6-item scale) typically yield lower variance and alpha values than longer, multidimensional scales. Considering the rapid applicability and clinical utility of the instrument in busy healthcare environments, these values are deemed robust enough to support the scale’s overall reliability and construct validity. Furthermore, internal consistency was verified, as the Cronbach’s alpha value exceeded the required threshold, demonstrating strong consistency within the instrument. These findings suggest that the scale can be reliably used in both research and clinical applications.

This study has some limitations as well. The cross-sectional design and the lack of a test–retest reliability assessment limit the evaluation of the scale’s stability over time. Prospective studies should administer the scale to the same participants at two different time points to report test–retest reliability. Furthermore, the sample’s exclusivity to a single country may limit the generalizability of the findings across diverse healthcare systems. Furthermore, this research was conducted using data collected from a single hospital, and the sample consisted mostly of highly educated, employed individuals without chronic illness. Therefore, the findings may not generalize to older people, individuals with lower digital literacy, or populations with chronic health conditions. Future research should recruit from multiple sites with more diverse sociodemographic and health profiles to improve external validity and representativeness of the findings.

Additionally, performing both EFA and CFA on the same study sample represents a methodological limitation that warrants acknowledgment. In scale development, evaluating construct validation steps on the same dataset may bring along chance factors. Although this approach is common in applied research, it may capitalize on sample-specific characteristics and may result in optimistic model fit estimates. An independent validation sample or a split-sample design could provide stronger evidence of the scale’s structural validity. However, obtaining an additional independent sample from the same hospital setting was not feasible due to practical constraints related to participant access, clinical workload, and ethical approval procedures. Therefore, prospective research should replicate and cross-validate the factor structure using an independent sample if possible. Moreover, prospective studies could employ common factor analysis and parallel analysis to identify latent structures. Accordingly, given the brief, highly compact, and unidimensional structure of this 6-item screening instrument, utilizing the same cohort was deemed practically and statistically viable. According to established psychometric literature [14,35], while splitting a sample or utilizing independent cohorts is ideal for complex multidimensional instruments, a single-sample approach provides robust statistical power for ultra-brief scales if the sample-size-to-item ratio is exceptionally high. In this study, the sample size (*n* = 304) corresponds to approximately 50 times the number of scale items, vastly exceeding the traditional threshold of 5 to 10 respondents per item. This high ratio minimizes cross-loading risks and ensures structural stability, thereby justifying the combined factor analyses within this specific methodological context. Nevertheless, future validation studies testing the eNUBS across diverse and independent clinical cohorts will further strengthen its external validity and generalizability.

However, the study’s large sample size and comprehensive factor analyses enhance the scientific reliability of the findings.

It is believed that such measurement tools will make significant contributions to the development of digital health policies, the enhancement of patient engagement, and the effective monitoring of the digital transformation of healthcare services.

## 5. Conclusions

The developed e-Nabız Usage Behavior Scale can serve as a valid and reliable instrument for evaluating individuals’ behavior regarding the e-Nabız system. The scale can also serve as a guide for clinical practice, primary healthcare services, and digital health research.

## Figures and Tables

**Table 1:** The Instrument Properties.

Items	Factor Loadings	Communality ($h^{2}$)	CITC
1	0.70	0.50	0.52
2	0.66	0.44	0.48
3	0.59	0.35	0.43
4	0.62	0.38	0.45
5	0.70	0.49	0.53
6	0.76	0.58	0.59
**Scale-Level Statistics**			
Eigenvalue (*λ*)	2.77		
Cronbach’s Alpha (*α*)	0.72		
Explained Variance (%)	46.21		

CITC: Corrected Item Total Correlation.

**Table 2:** E-Nabız Usage Behavior Scale.

	Read Each of the Following Items and Mark the Frequency That Best Suits You.	Never	Rarely	Occasionally	Frequently	Always
1	In the last 6 months, I have logged in to the e-Nabız system.					
2	I reviewed my prescriptions through e-Nabız.					
3	I made a hospital appointment using e-Nabız.					
4	I checked or changed my family physician information through the e-Nabız system.					
5	I use the e-Nabız application on my mobile device.					
6	To see my test or report results, I log in to e-Nabız					

## Data Availability

The original contributions presented in this study are included in the article. Further inquiries can be directed to the corresponding author.
